# Targeting chronic lymphocytic leukemia using CIGB-300, a clinical-stage CK2-specifc cell-permeable peptide inhibitor

**DOI:** 10.18632/oncotarget.1513

**Published:** 2013-12-13

**Authors:** Leila R. Martins, Yasser Perera, Paulo Lúcio, Maria G. Silva, Silvio E. Perea, João T. Barata

**Affiliations:** ^1^ Instituto de Medicina Molecular, Faculdade de Medicina, Universidade de Lisboa, Lisbon, Portugal;; ^2^ Centro de Ingeniería Genética y Biotecnología, Havana, Cuba;; ^3^ CEDOC, Faculdade de Ciências Médicas, FCM, Universidade Nova de Lisboa and Instituto Português de Oncologia, Lisbon, Portugal.

**Keywords:** Chronic Lymphocytic Leukemia, CLL, Casein kinase 2, CK2, CIGB-300, Signaling therapies

## Abstract

Chronic lymphocytic leukemia (CLL) remains an incurable malignancy, urging for the identifcation of new molecular targets for therapeutic intervention. CLL cells rely on overexpression and hyperactivation of the ubiquitous serine/threonine protein kinase CK2 for their viability *in vitro*. CIGB-300 is a cell-permeable selective CK2 inhibitor peptide undergoing clinical trials for several cancers. Here, we show that CIGB-300 promotes activation of the tumor suppressor PTEN and abrogates PI3K-mediated downstream signaling in CLL cells. In accordance, CIGB-300 decreases the viability and proliferation of CLL cell lines, promotes apoptosis of primary leukemia cells and displays antitumor efcacy in a xenograft mouse model of human CLL. Our studies provide pre-clinical support for the testing and possible inclusion of CK2 inhibitors in the clinical arsenal against CLL.

## INTRODUCTION

Despite significant improvements in treatment outcome in recent years [1, 2], chronic lymphocytic leukemia (CLL) – the most common leukemia in the Western world – remains incurable [3, 4]. In addition, a significant fraction of patients does not tolerate the aggressive protocols that may prolong overall survival [5]. Thus, further understanding of CLL biology and pathophysiology are mandatory for the identification of new molecular targets and the development of rational, more efficient therapies against this malignancy.

The ubiquitous serine/threonine protein kinase CK2 is frequently overexpressed in cancer, including several hematological neoplasms [6-10]. Recently, we and others have shown that leukemia cells from CLL patients display higher CK2 expression and activity than normal B cells, leading to inhibition of PTEN and activation of PI3K signaling pathway [9, 10], which is required for CLL cell survival [11-13].

The accumulating evidence that tumor cells commonly rely on CK2 for their maintenance [14-16] stimulated the quest for new classes of CK2 antagonists [17] and drove the development of CK2 inhibitors for clinical application in cancer [18, 19]. CIGB-300 is a cell-permeable peptide that modulates CK2 activity by binding to the phosphoacceptor site on CK2 targets [18]. CIGB-300 demonstrated a dose-dependent antiproliferative and proapoptotic effect in a variety of tumor cells [20]. In vivo, both local and systemic administration of CIGB-300 elicited significant antitumor effects in murine syngeneic cancers and human tumors xenografted in nude mice [21]. Most importantly, phase I clinical trials in cervical cancer showed tumor reduction, and CIGB-300 was safe and well tolerated [22].

In the studies reported here, we used for the first time CIGB-300 to pre-clinically evaluate the potential of CK2 inhibition in CLL treatment.

## RESULTS

### CIGB-300 activates PTEN and inhibits PI3K signaling pathway in CLL cells

**Figure 1:**
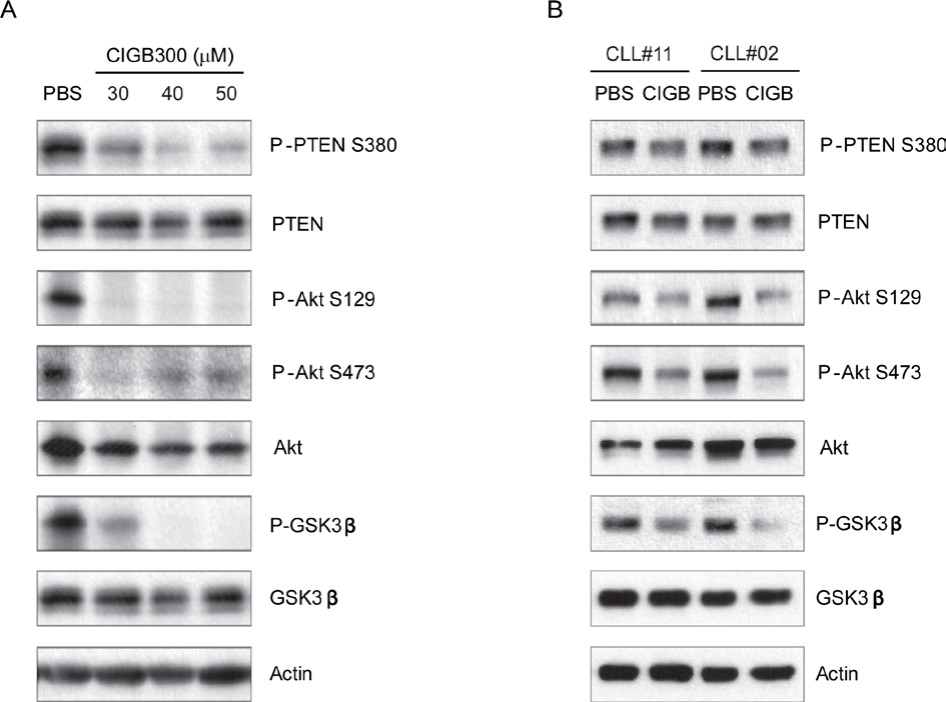
CIGB-300 inhibits PI3K signaling pathway CLL MO1043 cells were incubated with the indicated concentrations of CIGB-300 (A) and primary CLL cells were incubated with 12.5αM CIGB-300 (B). Cells were lysed after 2h and lysates were immunoblotted with antibodies against P-PTEN (S380), PTEN, P-Akt (S129), P-Akt (S473), Akt P-GSK3β (S9), GSK3, or actin as loading control.

Based on previous data showing that PI3K-mediated signals are required for survival of CLL cells in vitro [11, 13, 23], and that CK2 positively regulates PI3K pathway in CLL [9-11], we started by evaluating the impact of CIGB-300 on the interplay between CK2 and PI3K signaling. First, we confirmed that the peptide efficiently prevented phosphorylation of the direct CK2 target residue S129 on Akt/PKB (which leads to increased catalytic activity of already activated Akt) [24] in the MO1043 CLL cell line (Figure 1A) and in primary CLL cells (Figure 1B). Then, in accordance with results of other CK2 inhibitors, we found that incubation of CLL cells with CIGB-300

### CIGB-300 decreases the viability and proliferation of CLL cells and overcomes stromal support

**Figure 2:**
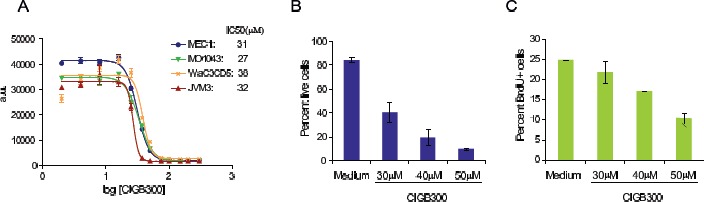
CIGB-300 decreases the viability and proliferation of CLL cell lines (A) CLL cell lines were incubated with increasing concentrations of CIGB-300 and IC50 was determined for each cell line at 72h with an AlamarBlue® assay. (B-C) MO1043 cells were cultured for 48h with the indicated CIGB-300 concentrations. Viability (B) and percentage of cells in S-phase (C) were assessed by FACS after annexin V/7-AAD staining and analysis of BrdU incorporation, respectively. Results indicate mean ± SD and are representative of 3 experiments analyzed.

**Figure 3:**
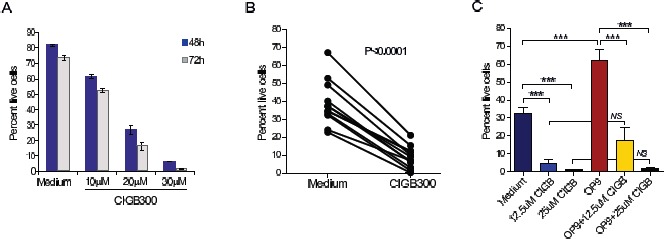
CIGB-300 decreases the viability of primary CLL cells in a dose- and time-dependent manner independently of stromal support. (A) Primary CLL cells were incubated with the indicated doses of CIGB-300 and viability was analyzed at 48 and 72h. Results are shown as mean ± SD of triplicates and are representative of 3 patients analyzed. (B) Primary CLL cells were cultured in medium alone or with 12.5αM CIGB (n=12) and cell viability was evaluated after 48h. (C) Primary CLL cells were cultured in medium alone or in the presence of OP9 cells with the indicated concentrations of CIGB-300. Results are shown as mean ± SD of triplicates and are representative of 4 patients analyzed. Cell viability was analyzed by FACS after annexin V/7-AAD staining. ***P<0.001.

Next, we sought to evaluate whether these molecular observations translated into functional impact on CLL cell viability and proliferation. The CLL cell lines MEC1, WaC3CD5, JVM3 and MO1043 were cultured with increasing concentrations of CIGB-300 and cytotoxicity was analyzed at 72h by Alamar blue assay. The IC50 of CIGB-300 on these cells ranged between 27 and 38µM, which is comparable to that of solid tumor cell lines displaying sensitivity to the inhibitor in vivo [18] (Figure 2A). A more detailed analysis revealed that both viability and proliferation of CLL cell lines decreased in a time-(not shown) and dose-dependent manner (Figure 2B,C and data not shown). The dose- and time-dependent impact of CIGB-300 extended to primary CLL samples collected from the peripheral blood of patients (Fig. 3A). Notably, 12.5µM CIGB-300 were sufficient to induce a dramatic decrease in viability in all CLL patient samples analyzed, even in poor prognosis cases such as those with 11q deletion (Fig. 3B and Table 1). To better define the therapeutic potential of the drug, we next assessed whether the effect of CIGB-300 on primary CLL cells is counteracted by stromal support. Culture with the murine stromal cell line OP9 enhanced the viability of primary CLL cells, as expected, but it did not reverse the pro-apoptotic effect of CIGB-300 in any of the CLL samples analyzed (Figure 3C).

**Table 1:** Clinical and biological characteristics of the patients analyzed

CLL #	Age (years)	Gender	CD38 status	Lymphocyte doubling time (months)	CD19+/CD5+ cells (%)	Clinical stage (Binet)	β2M (mg/l)	Cytogenetics
1	62	M	Positive	<12	92	A	3.99	normal
2	84	M	Positive	<12	96	C	4.43	del13
3	75	F	N/A	<12	61	B	3.85	normal
4	95	M	Negative	|>12	46	A	3.64	del13
5	47	M	Negative	|>12	65	B	2.04	Del13q, del11q
6	79	M	Negative	|>12	93	A	3.35	del13
7	59	M	Positive	<12	97	A	N/A	del13
8	87	M	Negative	|>12	50	A	3	del13
9	64	M	Negative	|>12	N/A	A	1.79	normal
10	74	F	Negative	|>12	N/A	B	2.93	normal
11	64	M	Positive	N/A	N/A	B	N/A	N/A
12*	62	M	Positive	N/A	90	C	3.86	tris12

* Apart from CLL patient #12, which finished treatment 3 months before sample collection, none of the patients analyzed received previous treatment.

### CIGB-300 delays CLL growth in vivo

**Figure 4:**
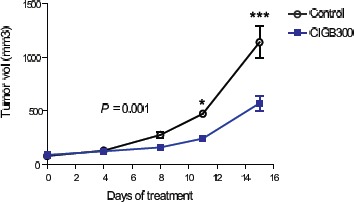
CK2 inhibition with CIGB-300 delays tumor growth in a subcutaneous CLL mouse model Previously irradiated Swiss nude mice were injected subcutaneously in the right flank with 5 x 10^6^ MO1043 cells. Three days after cell injection, mice were randomly distributed in 2 groups (n=6 per group): vehicle (control) and CIGB-300 (20mg/Kg, ip, 5 days + 2 days rest, every week, for 3 weeks). Tumor volume determined after treatment started. Statistical differences were evaluated using a two-way ANOVA test. * P<0.05 and ***P<0.001.

To further evaluate the clinical potential of CIGB-300, we xenotransplanted MO1043 cells subcutaneously into nude mice as described in the ‘Methods’. CIGB-300-treated mice presented a significant delay in tumor growth compared with the control group. The difference between groups was evident after approximately 1 week of treatment and became more significant with time (P<0.001, two-way ANOVA, Figure 4). Importantly, none of the mice showed weight loss or other toxicity-related symptoms throughout the experiment (not shown). These results suggest that targeting CK2 using CIGB-300 may be a valid therapeutic strategy in CLL.

## DISCUSSION

We and others previously showed that CK2 is a critical regulator of CLL cell viability [9-11]. However, those studies were conducted either after silencing of CK2 or using drugs that are not appropriate for in vivo administration [9-11] and therefore have limited clinical relevance. By contrast, in the present studies we explored for the first time the pre-clinical value of CIGB-300, a CK2 inhibitor that has already undergone clinical trials for other cancers and has demonstrated to be safe when used at clinically effective concentrations, with only mild to moderate toxicities [20].

We showed that treatment with CIGB-300 inhibited the viability and proliferation of CLL cell lines with IC50 values that are within a clinically suitable range. Moreover, CIGB-300 promoted apoptosis in all the primary CLL samples we analyzed, in a dose and time-dependent manner. Although anecdotal, it is noteworthy that this effect was evident even in one CLL case that presented del11q, which associates with poor prognosis and resistance to chemotherapy [26, 27]. Importantly, we also noted that stromal support by OP9 cells did not confer resistance against CK2 inhibition to primary CLL cells. These observations are in line with the in vivo effects of CIGB-300, which showed a clear delay in tumor development. Whether combination of CK2 inhibitors may synergize with novel targeted therapies [4] and contribute to the eradication of CLL is an exciting possibility that warrants further investigation.

Primary T-cell acute lymphoblastic leukemia [8] and CLL [9] cells display high levels of phosphorylation of PTEN (indicative of PTEN posttranslational inactivation) mediated by CK2. We found that CIGB-300 promoted PTEN activation and decreased the phosphorylation of Akt, a major PI3K downstream target. This notwithstanding, it should be underlined that PI3K signaling abrogation is probably just one of the mechanisms, albeit of critical importance, by which inhibition of CK2, a pleiotropic kinase that regulates numerous functions, may contribute to apoptosis of CLL cells. In fact, the pleiotropy of CK2 may be a fundamental cause for the addiction to CK2 activity displayed by tumor cells, and it is a key reason why, given the observations that CK2 inhibition does not result in intolerable side-effects, CK2 is a very promising therapeutic target in the context of cancer in general and CLL in particular.

In summary, our current data using CIGB-300, together with our parallel study demonstrating the pre-clinical efficacy of the CK2 small molecule inhibitor CX- 4945 [28], constitute strong evidence for the therapeutic potential of CK2 inhibition in the context of CLL and pave the way towards the clinical evaluation of these and other CK2 antagonists in this malignancy.

## METHODS

### Patient samples

Peripheral blood (PB) samples from CLL patients were collected in accordance with the Declaration of Helsinki after informed consent and ethical approval of Instituto Português de Oncologia (Lisbon, Portugal). Table 1 summarizes the clinical and biological characteristics of the patients analyzed. CLL cells were isolated from PB using RosetteSep human B cell enrichment cocktail (StemCell Technologies) as indicated by the manufacturer. The purity of CLL cells was always higher than 90%, as evaluated by staining with anti-CD5, CD3 and CD19 fluorochrome-conjugated antibodies (eBioscience) followed by flow cytometry analysis using a FACSCanto cytometer (Becton Dickinson).

### Cell culture and viability analysis

CLL cells were cultured as described [9, 28], and treated with CIGB-300 where specified. Viability was determined by flow cytometry after staining with Annexin V-APC (eBioscience) and 7-AAD (Becton Dickinson) [9]. Cytotoxicity was determined using AlamarBlue assay. Briefly, cell lines were seeded in fat bottom 96-wells plates under the culture conditions described above and indicated concentrations of CIGB-300 were added. Following 3 days of incubation with CIGB-300, Alamar Blue (Life Technologies) was added to cell cultures in an amount equal to 10% of the culture volume. Cells were further incubated at 37°C, 5% CO2 for 2-4 hours. Fluorescence was measured at wavelengths of 530nm excitation and 580nm emission in an Infinite M200 plate reader (Tecan).

### In vivo experiments

Eight week-old swiss nude mice, full-body irradiated with 600cGy the day before, were injected subcutaneously in the right flank with 5x106 MO1043 cells resuspended in 100µL of 50% Matrigel (Becton Dickinson) in PBS. At day 3, all mice presented palpable tumors (100-150mm3) and were randomly assigned into two groups (n=6 per group) to receive CIGB-300 intraperitoneal (ip) at 20 mg/ kg or vehicle, once a day, for 5 consecutive days every week, for a total of 3 weeks. Mice were monitored daily and weighed frequently to determine possible treatment induced toxicities. Tumors were measured every other day with a caliper and tumor volume was calculated (volume = length x width^2^/2). Mice were sacrificed when tumor reached 1500mm3. Housing and treatment was in accordance with EU guidelines and institutional ethics committee approval.

### Immunoblot

Immunoblotting was performed [29] using antibodies against: actin (Santa Cruz Biotechnology), P-Akt (S129) (ABGent), P-Akt (S473), Akt, P-PTEN (S380), PTEN, P-GSK3β (S9) and GSK3 (Cell Signaling Technology).

### Statistical analysis

Differences between mean values were evaluated using 2-tailed Student's t test. Differences in tumor growth were determined by a two-way ANOVA. Significance was set for P<0.05. All analyses were performed using GraphPad Prism (GraphPad Software).

## References

[1] Tam CS, O'Brien S, Wierda W, Kantarjian H, Wen S, Do KA, Thomas DA, Cortes J, Lerner S, Keating MJ (2008). Long-term results of the fludarabine, cyclophosphamide, and rituximab regimen as initial therapy of chronic lymphocytic leukemia. Blood.

[2] Cheng MM, Goulart B, Veenstra DL, Blough DK, Devine EB (2012). A network meta-analysis of therapies for previously untreated chronic lymphocytic leukemia. Cancer Treat Rev.

[3] Isfort S, Cramer P, Hallek M (2012). Novel and emerging drugs for chronic lymphocytic leukemia. Curr Cancer Drug Targets.

[4] Schnaiter A, Stilgenbauer S (2010). Refractory chronic lymphocytic leukemia--new therapeutic strategies. Oncotarget.

[5] Gribben JG, O'Brien S (2011). Update on therapy of chronic lymphocytic leukemia. J Clin Oncol.

[6] Piazza F, Manni S, Ruzzene M, Pinna LA, Gurrieri C, Semenzato G (2012). Protein kinase CK2 in hematologic malignancies: reliance on a pivotal cell survival regulator by oncogenic signaling pathways. Leukemia.

[7] Barata JT (2011). The impact of PTEN regulation by CK2 on PI3K-dependent signaling and leukemia cell survival. Adv Enzyme Regul.

[8] Silva A, Yunes JA, Cardoso BA, Martins LR, Jotta PY, Abecasis M, Nowill AE, Leslie NR, Cardoso AA, Barata JT (2008). PTEN posttranslational inactivation and hyperactivation of the PI3K/Akt pathway sustain primary T cell leukemia viability. J Clin Invest.

[9] Martins LR, Lucio P, Silva MC, Anderes KL, Gameiro P, Silva MG, Barata JT (2010). Targeting CK2 overexpression and hyperactivation as a novel therapeutic tool in chronic lymphocytic leukemia. Blood.

[10] Shehata M, Schnabl S, Demirtas D, Hilgarth M, Hubmann R, Ponath E, Badrnya S, Lehner C, Hoelbl A, Duechler M, Gaiger A, Zielinski C, Schwarzmeier JD, Jaeger U (2010). Reconstitution of PTEN activity by CK2 inhibitors and interference with the PI3-K/Akt cascade counteract the antiapoptotic effect of human stromal cells in chronic lymphocytic leukemia. Blood.

[11] Martins LR, Lucio P, Silva MC, Gameiro P, Silva MG, Barata JT (2011). On CK2 regulation of chronic lymphocytic leukemia cell viability. Mol Cell Biochem.

[12] Cuni S, Perez-Aciego P, Perez-Chacon G, Vargas JA, Sanchez A, Martin-Saavedra FM, Ballester S, Garcia-Marco J, Jorda J, Durantez A (2004). A sustained activation of PI3K/NF-kappaB pathway is critical for the survival of chronic lymphocytic leukemia B cells. Leukemia.

[13] Plate JM (2004). PI3-kinase regulates survival of chronic lymphocytic leukemia B-cells by preventing caspase 8 activation. Leuk Lymphoma.

[14] Ruzzene M, Pinna LA (2010). Addiction to protein kinase CK2: a common denominator of diverse cancer cells?. Biochim Biophys Acta.

[15] Yoo JY, Lim BJ, Choi HK, Hong SW, Jang HS, Kim C, Chun KH, Choi KC, Yoon HG (2013). CK2-NCoR signaling cascade promotes prostate tumorigenesis. Oncotarget.

[16] Turowec JP, Vilk G, Gabriel M, Litchfield DW (2013). Characterizing the convergence of protein kinase CK2 and caspase-3 reveals isoform-specific phosphorylation of caspase-3 by CK2alpha': implications for pathological roles of CK2 in promoting cancer cell survival. Oncotarget.

[17] Moucadel V, Prudent R, Sautel CF, Teillet F, Barette C, Lafanechere L, Receveur-Brechot V, Cochet C (2011). Antitumoral activity of allosteric inhibitors of protein kinase CK2. Oncotarget.

[18] Perea SE, Reyes O, Puchades Y, Mendoza O, Vispo NS, Torrens I, Santos A, Silva R, Acevedo B, Lopez E, Falcon V, Alonso DF (2004). Antitumor effect of a novel proapoptotic peptide that impairs the phosphorylation by the protein kinase 2 (casein kinase 2). Cancer Res.

[19] Siddiqui-Jain A, Drygin D, Streiner N, Chua P, Pierre F, O'Brien SE, Bliesath J, Omori M, Huser N, Ho C, Proffitt C, Schwaebe MK, Ryckman DM, Rice WG, Anderes K (2010). CX-4945, an orally bioavailable selective inhibitor of protein kinase CK2, inhibits prosurvival and angiogenic signaling and exhibits antitumor efficacy. Cancer Res.

[20] Perea SE, Reyes O, Baladron I, Perera Y, Farina H, Gil J, Rodriguez A, Bacardi D, Marcelo JL, Cosme K, Cruz M, Valenzuela C, Lopez-Saura PA, Puchades Y, Serrano JM, Mendoza O (2008). CIGB-300, a novel proapoptotic peptide that impairs the CK2 phosphorylation and exhibits anticancer properties both in vitro and in vivo. Mol Cell Biochem.

[21] Perera Y, Farina HG, Hernandez I, Mendoza O, Serrano JM, Reyes O, Gomez DE, Gomez RE, Acevedo BE, Alonso DF, Perea SE (2008). Systemic administration of a peptide that impairs the protein kinase (CK2) phosphorylation reduces solid tumor growth in mice. Int J Cancer.

[22] Perea SE, Baladron I, Garcia Y, Perera Y, Lopez A, Soriano JL, Batista N, Palau A, Hernandez I, Farina H, Garcia I, Gonzalez L, Gil J, Rodriguez A, Solares M, Santana A (2011). CIGB-300 a synthetic peptide-based drug that targets the CK2 phosphoaceptor domain. Translational and clinical research. Mol Cell Biochem.

[23] Ringshausen I, Schneller F, Bogner C, Hipp S, Duyster J, Peschel C, Decker T (2002). Constitutively activated phosphatidylinositol-3 kinase (PI-3K) is involved in the defect of apoptosis in B-CLL: association with protein kinase Cdelta. Blood.

[24] Di Maira G, Salvi M, Arrigoni G, Marin O, Sarno S, Brustolon F, Pinna LA, Ruzzene M (2005). Protein kinase CK2 phosphorylates and upregulates Akt/PKB. Cell Death Differ.

[25] Stambolic V, Suzuki A, de la Pompa JL, Brothers GM, Mirtsos C, Sasaki T, Ruland J, Penninger JM, Siderovski DP, Mak TW (1998). Negative regulation of PKB/Akt-dependent cell survival by the tumor suppressor PTEN. Cell.

[26] Kay NE, O'Brien SM, Pettitt AR, Stilgenbauer S (2007). The role of prognostic factors in assessing ‘high-risk’ subgroups of patients with chronic lymphocytic leukemia. Leukemia.

[27] Austen B, Skowronska A, Baker C, Powell JE, Gardiner A, Oscier D, Majid A, Dyer M, Siebert R, Taylor AM, Moss PA, Stankovic T (2007). Mutation status of the residual ATM allele is an important determinant of the cellular response to chemotherapy and survival in patients with chronic lymphocytic leukemia containing an 11q deletion. J Clin Oncol.

[28] Martins LR, Lucio P, Melao A, Antunes I, Cardoso BA, Stansfield R, Bertilaccio MT, Ghia P, Drygin D, Silva MG, Barata JT (2013). Activity of the clinical-stage CK2-specifc inhibitor CX-4945 against chronic lymphocytic leukemia. Leukemia.

[29] Silva A, Jotta PY, Silveira AB, Ribeiro D, Brandalise SR, Yunes JA, Barata JT (2010). Regulation of PTEN by CK2 and Notch1 in primary T-cell acute lymphoblastic leukemia: rationale for combined use of CK2- and gamma-secretase inhibitors. Haematologica.

